# Relevance of *LIG4* gene polymorphisms with cancer susceptibility: Evidence from a meta-analysis

**DOI:** 10.1038/srep06630

**Published:** 2014-10-15

**Authors:** Shang Xie, Xiao-Feng Shan, Kun Shang, Hui Xu, Jing He, Zhi-Gang Cai

**Affiliations:** 1 Department of Oral and Maxillofacial Surgery, Peking University School and hospital of Stomatology, Beijing 100081, China; 2 Department of Nuclear Medicine, Xuanwu Hospital, Capital Medical University, Beijing 100053, China; 3 State Key Laboratory of Oncology in South China, Department of Experimental Research, Collaborative Innovation Center for Cancer Medicine, Sun Yat-Sen University Cancer Center, Guangzhou 510060, Guangdong, China

## Abstract

Polymorphisms of *LIG4* gene may influence DNA repair ability, thus altering the genetic stability and resulting in carcinogenesis. A growing number of studies have investigated the relevance of *LIG4* T9I (rs1805388) and D501D (rs1805386) polymorphisms with cancer risk, however, the results are conflicting. To obtain a comprehensive conclusion, we searched relevant literatures from PubMed, Web of Science, Ovid and Embase databases on May 15, 2014 and performed a meta-analysis. In this meta-analysis, a total of 17 articles were included. Of them, there were 15 studies with 5873 cases and 5771 controls for rs1805388 and 6 studies with 4161 cases and 4881 controls for rs1805386. Overall, our results suggested that there was no obvious relevance of *LIG4* T9I polymorphism with cancer susceptibility. However, in subgroup analysis, we found the *LIG4* T9I was associated with a slightly decreased cancer risk among Caucasians. As to the rs1805386, the genetic variant had no significant association with cancer risk. In conclusion, despite several limitations, this meta-analysis suggested that *LIG4* T9I genetic variant is associated with a decreased risk of cancer among Caucasians, however, the rs1805386 gene polymorphism is not a risk factor of cancer.

Cancer is one of the most common causes of death in the world and results in a serious problem to global health^1^,^2^. Despite advances in treatment for cancer, the prognosis remains unsatisfied^3^. Thus, exploring the methods of early detection and prevention are indispensable. Currently, gene-environment interactions have been thought to be main determinant of individual risk for diseases including cancer^4^. Genes decide the susceptibility of individual to environment and environmental factors often damage the DNA in turn. If the host DNA repair system does not perform their functions well in repairing such destructive DNA, it might alter the stability of genome and lead to carcinogenesis. Thus, the DNA repair ability plays a critical role in maintaining the stability of human genome.

*LIG4* gene, located on human chromosomes 13q33-34, encodes an ATP-dependent DNA ligase that joins single-strand breaks in a double-stranded polydeoxynucleotide in an ATP-dependent reaction^5^. The DNA ligase IV is the crucial enzyme to for completing the non-homologous end joining (NHEJ) by forming a complex together with X-ray repair cross complementing protein 4 (XRCC4) for a final ligation of the break in an ATP-dependent step^6^,^7^. Therefore, the loss or variant of *LIG4* is supposed to contribute to genomic instability and tumorigenesis.

According to supposition mentioned above, we reviewed the related studies concerning of *LIG4* variants and susceptibility of tumor. Then, we found that numerous studies investigated this issue, however, the conclusions are inconsistent. To obtain a comprehensive conclusion, we carried out a meta-analysis to systematically evaluate the relevance of *LIG4* genetic variants with susceptibility of cancer.

## Results

### Identification of relevant studies

**Figure 1:**
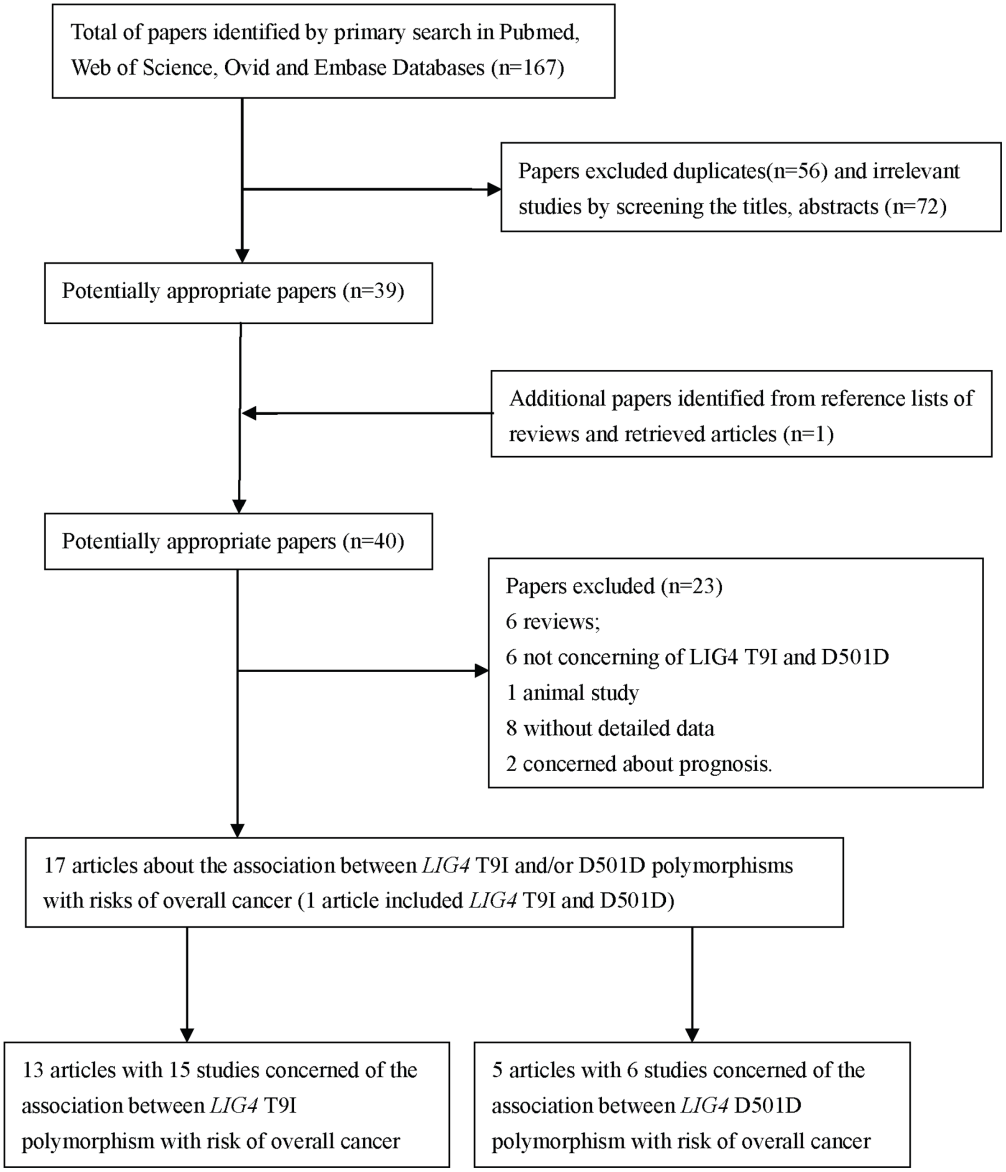
Flow diagram of included/excluded studies.

A total of 167 papers were indentified from the databases as we described above. After deleting the duplications, 111 papers were left. Then we estimated the rest articles and 72 articles were discarded because of irrelevance with this issue. And one other potential eligible paper was obtained by screening the references of reviews. Of the remains 40 papers, one article was excluded for animal study^8^; six papers were reviews^9^,^10^,^11^,^12^,^13^,^14^. Following this, two papers were concerned with prognosis^15^,^16^, eight articles without detailed data for further evaluation^17^,^18^,^19^,^20^,^21^,^22^,^23^,^24^. Besides, six papers estimated *LIG4* polymorphism but not T9I or D501D^25^,^26^,^27^,^28^,^29^,^30^. Finally, a total of 17 studies with case-control design met the inclusion and exclusion criteria in this meta-analysis^31^,^32^,^33^,^34^,^35^,^36^,^37^,^38^,^39^,^40^,^41^,^42^,^43^,^44^,^45^,^46^,^47^. Of these 17 articles (one article included both rs1805386 and rs1805388), there were five articles with six studies for rs1805386, 13 articles with 15 studies for rs1805388. The flow diagram of searching process was shown in Figure 1.

### Characteristics of included studies

Of these included articles, one article^42^ with three caner types were separated as three independent studies and three articles^35^,^39^,^45^ concluded rs1805386 and rs1805388. Noticeably, in the relevant articles, two articles^19^,^24^ did not find the polymorphism of rs1805386 and another article^45^ did supply with insufficient data for rs1805386 but for rs1805388. Thus, in the end, there were five articles with six studies for rs1805386, 13 articles with 15 studies for rs1805388 polymorphism.

Then, we established a database concerning of the information extracted from each included paper. Summaries of these studies were presented in Table 1 which included the first author's surname, publication year, ethnicity, country, number and characteristics of cases and controls, and other relevant information.

### Meta-analysis results

#### rs1805386

We found that there was no obvious relevance of *LIG4* D501D variants with overall cancer risk (homozygous: OR = 0.97, 95% CI = 0.59–1.59, *P* = 0.907; recessive: OR = 0.96, 95% CI = 0.88–1.06, *P* = 0.434; dominant: OR = 0.99, 95% CI = 0.61–1.60, *P* = 0.952; allele: OR = 0.95, 95% CI = 0.87–1.03, *P* = 0.229) (Table 2). In the subgroup analysis stratified by cancer types, no statistically significant relations were found for breast cancer and ovarian cancer (The data were not shown).

#### rs1805388

**Figure 2:**
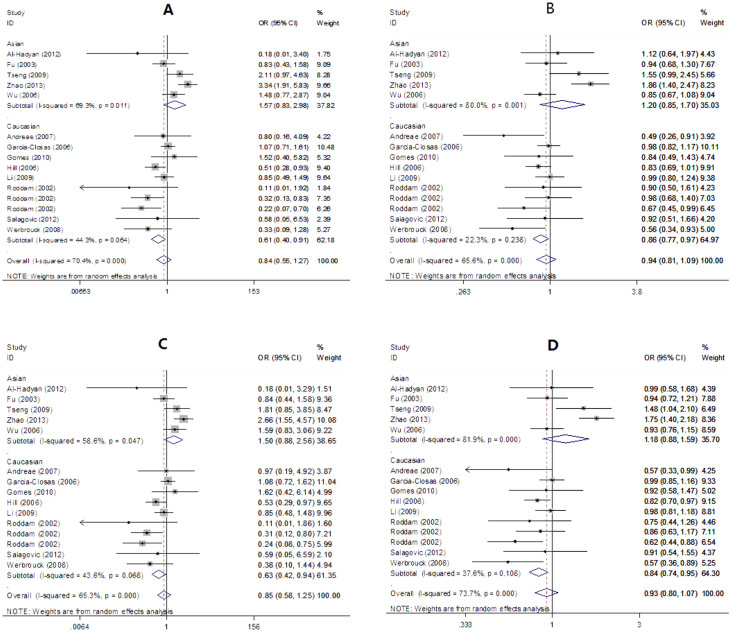
Forest plot for overall cancer risk associated with *LIG4* T9I polymorphism ((A): homozygous model; (B): recessive model; (C): dominant model; (D): allele model. subgroup analysis by ethnicity).

As shown in Table 2 and Figure 2, there was no relevance of *LIG4* T9I variants with overall cancer risk (homozygous: OR = 0.84, 95% CI = 0.55–1.27, *P* = 0.401; recessive: OR = 0.94, 95% CI = 0.81–1.09, *P* = 0.434; dominant: OR = 0.85, 95% CI = 0.58–1.25, *P* = 0.410; allele: OR = 0.93, 95% CI = 0.80–1.07, *P* = 0.306). However, in the subgroup analysis, a statistically significant association was found among Caucasians (homozygous: OR = 0.61, 95%CI = 0.40–0.91, *P* = 0.016; recessive: OR = 0.86, 95% CI = 0.77–0.97, *P* = 0.016; dominant: OR = 0.63, 95% CI = 0.42–0.94, *P* = 0.023; allele: OR = 0.84, 95%CI = 0.74–0.95, *P* = 0.004).

### Heterogeneity and sensitivity analysis

Heterogeneities were observed among several studies for *LIG4* D501D polymorphism and overall cancer susceptibility (homozygous: *P* = 0.044; dominant: *P* = 0.048;) and T9I (homozygous: *P* < 0.001; recessive: *P* < 0.001; dominant: *P* < 0.001; allele: *P* < 0.001). Thus, we selected the random-effect models to generate pooled ORs and corresponding 95% CIs for these models. On the other hand, no heterogeneity was observed among the other two models for D501D (recessive: *P* = 0.520; allele: *P* = 0.218) and the fixed-effect models were performed to generate ORs and 95% CIs for them. The sensitivity analysis suggested that no obvious changes were observed for the pooled ORs when single investigation was excluded respectively (data were not shown).

### Publication bias

The shape of the funnel plot showed that no evidence of asymmetry was observed in the current meta-analysis by Egger's test for T9I (homozygous: *P* = 0.530; recessive: *P* = 0.919; dominant: *P* = 0.482; allele: *P* = 0.585) and D501D (homozygous: *P* = 0.501; recessive: *P* = 0.073; dominant: *P* = 0.499; allele: *P* = 0.451), which suggested that there were no significant publication bias among all these studies.

### Trial sequential analysis (TSA)

**Figure 3:**
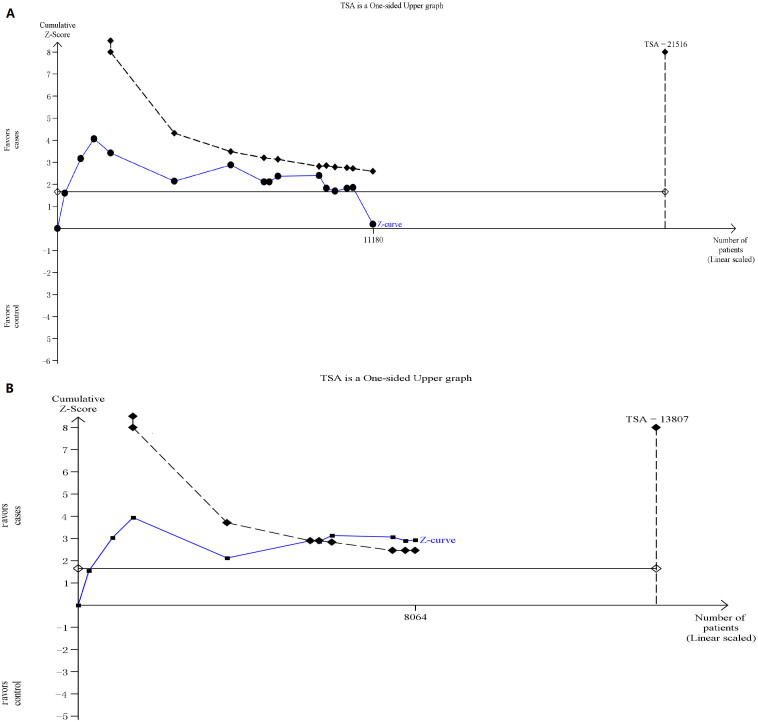
(A): The required information size to demonstrate the relevance of rs1805388 gene polymorphisms with cancer susceptibility; (B): The required information size to demonstrate the relevance of rs1805388 gene polymorphisms with risk of cancer among Caucasians. The solid blue line is the cumulative Z-curve. The dashed inward-sloping line to the left represents the trial sequential monitoring boundaries.

Fifteen trials (11180 subjects) were used to investigate the relevance of rs1805388 gene polymorphism with cancer susceptibility. Using the TSA (taking the data of dominant model for example), the required information size is 21516 subjects to demonstrate the issue (Figure 3A). Until now, the cumulative z-curve has not crossed the trial monitoring boundary before reaching the required information size, indicating that the cumulative evidence is insufficient and further trials are necessary. However, the cumulative z-curve crossed with TSA monitoring boundary when we performed the sub-analysis based on the ethnicity, confirming that rs1805388 is associated with a slightly decreased cancer risk among Caucasians and further relevant trials are unnecessary (Figure 3B). As for rs1805386, we chose the data of four models to perform TSA. The cumulative z-curve have crossed with TSA monitoring boundaries before the required information size is reached, indicating that the rs1805386 polymorphism is not a risk factor of cancer and no further trials are necessary (figures were not shown).

## Discussion

Genomic instability is an outstanding characteristic of cancer^48^. Numerous studies have demonstrated that tumor suppressor genes play a significant role in DNA double-strand break (DSB) repair and in maintenance of genomic stability; correspondingly, loss or mutation of such repair genes results in a shifty susceptibility for malignancies.

In eukaryotes, homologous recombination and NHEJ are two major pathways for DNA DSB repair and the latter is predominantly way in mammalian cells^49^. In the NHEJ process, LIG4, as a major functional protein, forms a complex with XRCC4 to perform the final rejoining step of NHEJ. Thus, the genetic variant of LIG4 might alter the repair capacity of DSB.

Previous investigation had demonstrated that polymorphisms in DNA repair genes might alter DNA repair capacity and thus contribute to cancer risk^50^. In 2002, Kuschel et al.^40^ found that *LIG4* D501D polymorphism was associated with a decrease in breast cancer risk and Roddam et al.^42^ found that *LIG4* T9I polymorphism may modulate predisposition to multiple myeloma. After these discoveries, a mass of investigations were performed to estimate the association between *LIG4* T9I and D501D polymorphisms and the susceptibility of various cancers. To our puzzled, the results are conflicting. Thus, we performed a meta-analysis to obtain a comprehensive conclusion.

To our knowledge, this is the first time to systematically estimate the associations between *LIG4* T9I and D501D polymorphisms and susceptibility of overall cancer. In this study, we found that rs1805386 and rs1805388 genetic variants had no relevance with overall cancer risk in homozygous, recessive, dominant and allele models. To further investigate the associations, we performed the subgroups analysis based on ethnicity and cancer types and found that rs1805388 variant is a decreased risk of cancers among Caucasians.

In order to make the conclusion more credible, we performed the publication bias analysis and sensitivity analysis according to Cochrane protocol. Funnel plots suggested that no obvious publication bias was observed. The sensitivity analysis indicated that the results are robust and no single study yield to obvious effects on the pooled ORs and the corresponding CIs. Besides, we performed the TSA and the results of TSA showed that the conclusions in this meta-analysis are robust.

However, several limitations in this meta-analysis should be noticed. Firstly, several studies had small sample sizes which might lessen the statistical power. Secondly, the heterogeneity was existed and thus we performed the random-effects model to obtain the wider CIs, which might weaken the reliability of conclusions. Thirdly, our results were based on unadjusted assessment of ORs, which might influence the results. Fourthly, we did not search the unpublished studies, which might miss the relevant studies. Besides, all data included in this study were from published investigations which were based on the current marker identification method of the ‘one-step-clustering'. This approach might tend to be ‘passenger signals' instead of ‘drivers' and bury the ‘real' cancer gene, which made the results less robust and accurate^51^,^52^. Based on the limitations mentioned above, the results should be considered with caution.

Overall, in spite of these limitations, this analysis reached a precise conclusion that *LIG4* D501D polymorphism has no obvious relevance with cancer risk and individual with *LIG4* T9I genetic variant has a decrease cancer risk among Caucasians. With the development of research methods, future studies focusing on a combinatorial use of the polygene markers and integrative network modules analysis, are necessary to make the conclusions more comprehensive.

## Methods

### Search strategy

We searched the PubMed, Web of Science, Ovid and Embase databases without language limitations for all related papers using the following searching strategies: 1) *LIG4* or *LIG 4* or *ligase IV*, 2) polymorphism or variant or variation or allele or genotype, and 3) cancer or carcinoma or tumor or neoplasm. And the last research was updated on May 15, 2014. All searched studies were screened and their references were retrieved to obtain other related articles. Then we downloaded the relevant papers and further screened to identify potentially eligible studies.

### Inclusion/exclusion criteria

Studies included in this studies had to meet the following inclusion criteria: (1) estimating the relevance of *LIG4* polymorphisms (rs1805386 and rs1805388) and cancer susceptibility; (2) case-control design; (3) sufficient data provided to assess odds ratios (ORs) and the corresponding confidence intervals (CIs); (4) when multiple publication from a particular research group reported data from overlapping samples, the study reporting the largest or latest dataset was included. Exclusion criteria: (1) review articles; (2) case reports, or case-only studies; (3) studies that estimated the risk of prognosis.

### Data extraction

All data were independently reviewed and extracted from the included papers by two authors (S.X. and J.H.). Disagreements were solved by full discussion until a consensus was reached. The following characteristics were collected from each study: first author's surname, year of publication, ethnicity, country, cancer type, sample size, control source, matching contents, the Hardy-Weinberg equilibrium, genotype methods and genetic distribution of cases and controls. The subgroup analysis was performed by cancer type and ethnicity.

### Statistical analysis

All the data management and analysis for this meta-analysis were performed with STATA 11.0 software (Stata corporation, College Station, TX). The strength of association between rs1805386 and rs1805388 polymorphisms and cancer susceptibility was estimated by calculating OR with the corresponding 95% CI. In order to calculate heterogeneity of studies, the Chi-Square test was used and significance was set at *P* value less than 0.05 level^53^. If the study was found to be heterogeneous (*P* > 0.10 for the Q-test), the fixed-effects model (the Mantel-Haenszel method) was performed to calculate the combined OR^54^. Otherwise, a random effect model (the DerSimonian and Laird method) was used to estimate the pooled OR^55^. In addition, the heterogeneity was also quantified with *I^2^* statistics. The *I^2^* value ranges from 0 to 100% and a larger *I^2^* value indicating a greater heterogeneity^56^. The funnel plot was used to test the potential publication bias, and the funnel plot asymmetry was estimated by Egger's linear regression^57^. Sensitivity analyses were performed to identify the influence of the each study on the combined ORs and 95% CI.

### Trial sequential analysis (TSA)

According to Cochrane Handbook for systematic reviews of interventions, meta-analyses and systematic reviews are considered to be the best available evidence if all available trials are included. However, ‘the best available evidence' might not be equal to ‘strong evidence' or ‘sufficient evidence'. It is well-known that meta-analysis may cause random errors in the series of sparse data and reduplicative testing on accumulating data. Based on these problems mentioned above, we applied the TSA to minimize the random errors and increase the robustness of conclusions^58^,^59^. In our study, we planned to calculate the required information size and estimate how many patients would be necessary to make a robust conclusion^58^. The required information size was based on the assumption of a plausible relative risk of 10% with low risk bias, and we adopted the risks for a type I error (α) of 5%, a type II error (β) of 20%^58^. Based on required information size and risk for type I and type II errors TSA monitoring boundaries were built. If a TSA monitoring boundary is crossed with Z-curve before the required information size is reached, robust evidence might have been confirmed and further trials are unnecessary. Otherwise, it is necessary to continue doing trials.

## Author Contributions

All authors contributed significantly to this work. Z.-G.C. designed and revised the article. J.H. collected articles, summarized data and revised this article. S.X. collected articles, summarized data, did statistical work and drafted the manuscript. X.-F.S. collected articles, summarized data and did statistical work. H.X. and K.S. provided collected data and summarized information in part. All authors reviewed this manuscript and approved the final draft.

## Figures and Tables

**Table 1:** Characteristics of studies included in this meta-analysis

Surname	Year	Country	Ethnicity	Cancer type	Control source	Genotyping methods	Cases/Control	D. Case	D. Control	HWE
rs1805388										
Roddam	2002	UK	Caucasian	acute lymphoblastic leukemia	NA	PCR	70/220	0/21/49	13/58/149	0.03
Roddam	2002	UK	Caucasian	lymphoma	NA	PCR	362/220	7/108/247	13/58/149	0.03
Roddam	2002	UK	Caucasian	multiple myeloma	NA	PCR	269/220	4/61/204	13/58/149	0.03
Fu	2003	China	Asian	breast cancer	socioeconomic status	MassARRAY	253/376	16/100/137	28/150/198	0.955
Garcia-Closas	2006	USA	Caucasian	breast cancer	Age	Taqman	1316/1043	57/339/920	42/277/724	0.199
Hill	2006	USA	Caucasian	non-Hodgkin lymphoma	Age, gender	Taqman	1110/931	18/300/792	28/275/628	0.75
Wu	2006	China	Asian	bladder cancer	Age, gender	PCR-RFLP	606/593	24/168/414	15/194/384	0.099
Andreae	2007	Germany	Caucasian	acute lymphoblastic leukemia	Age, gender	DHPLC	107/104	3/19/85	3/33/68	0.673
Werbrouck	2008	Belgium	Caucasian	head and neck cancer	Age, gender	PCR-RFLP	152/157	3/30/119	8/44/105	0.242
Tseng	2009	Taiwan	Asian	lung cancer	Age, gender	PCR	149/152	20/62/67	12/55/85	0.464
Li	2009	USA	Caucasian	pancreatic cancer	Age, gender	Taqman	723/776	23/197/503	29/208/539	0.117
Gomes	2010	Portugal	Caucasian	thyroid cancer	Age, gender	Real-Time PCR	109/217	4/22/83	5/54/158	0.879
Al-Hadyan	2012	Saudi Arabia	Asian	head and neck cancer	Age	PCR	156/251	0/24/132	4/31/216	0.029
Salagovic	2012	Slovakia	Caucasian	lymphoma	NA	PCR	107/127	1/26/80	2/32/93	0.688
Zhao	2013	China	Asian	glioma	Age, gender	PCR	384/384	49/172/163	20/142/222	0.659
rs1805386										
Kuschel	2002	Germany	Caucasian	breast cancer	NA	PCR	1440/2016	20/322/914	50/492/1248	0.857
Han	2004	USA	Caucasian	breast cancer	Age, BMI	Taqman	977/1266	22/274/681	32/360/874	0.48
Garcia-Closas	2006	USA	Caucasian	breast cancer	Age	Taqman	1338/1057	55/379/904	34/309/714	0.934
Jakubowska	2010	Germany	Caucasian	breast cancer	NA	PCR	319/290	112*/207	94*/196	NA
Jakubowska	2010	Germany	Caucasian	ovarian cancer	NA	PCR	145/280	54*/91	92*/188	NA
Assis	2010	Portugal	Caucasian	ovarian cancer	NA	Real-Time PCR	126/198	7/36/83	5/60/133	0.562

NA: not available; D.: distribution;

*: homozygous variants + heterozygous variant (CC + TC); HWE: Hardy-Weinberg Equilibrium.

**Table 2:** Relevance of rs1805388 and rs1805386 with cancer risk in this Meta-analysis

		Homozygous			Recessive			Dominant			Allele		
Variables	No of individuals	OR (95%CI)	*P*^het^	*I^2^* (%)	OR (95% CI)	*P*^het^	*I^2^* (%)	OR(95% CI)	*P*^het^	*I^2^* (%)	OR (95% CI)	*P*^het^	*I^2^* (%)
For T9I		TT vs. CC			(TT + TC) vs. CC			TT vs. (TC + CC)			T vs. C		
All	5873/5771	0.84 (0.55–1.27)	0.000	0.704	0.94 (0.81–1.09)	0.000	65.6	0.85 (0.58–1.25)	0.000	65.3	0.93 (0.80–1.07)	0.000	73.7
Ethnicity													
Asian	1941/1991	1.57 (0.83–2.98)	0.011	0.693	1.20 (0.85–1.70)	0.001	80.0	1.50 (0.88–2.56)	0.047	58.6	1.18 (0.88–1.59)	0.000	81.9
Caucasian	3932/3780	0.61 (0.40–0.91)	0.064	0.443	0.86 (0.77–0.97)	0.238	22.3	0.63 (0.42–0.94)	0.068	43.6	0.84 (0.74–0.95)	0.108	37.6
For D501D		CC vs. TT			(CC + CT) vs. TT			CC vs. (CT + TT)			C vs. T		
All	4161/4881	0.97 (0.59–1.59)	0.044	63.1	0.96 (0.88–1.06)	0.520	0.0	0.99 (0.61–1.60)	0.048	62.1	0.95 (0.87–1.03)	0.218	32.4
